# Mismatch negativity of preschool children at risk of developing mental health problems

**DOI:** 10.1002/npr2.12168

**Published:** 2021-02-19

**Authors:** Toshiya Aoi, Takashi X. Fujisawa, Shota Nishitani, Akemi Tomoda

**Affiliations:** ^1^ Department of Child Development United Graduate School of Child Development Osaka University Kanazawa University Hamamatsu University School of Medicine Chiba University and University of Fukui Osaka Japan; ^2^ Department of Nursing Faculty of Health Science Fukui Health Science University Fukui Japan; ^3^ Research Center for Child Mental Development University of Fukui Fukui Japan

**Keywords:** event‐related potential, mismatch negativity, preschool children, strengths and difficulties questionnaire

## Abstract

This study examined the relationship between mismatch negativity (MMN) during the passive oddball task and clinical assessment using a behavioral scale in nonclinical preschool children to identify neurobiological endophenotypes associated with the risk of developing mental health problems. We assessed the risk of developing mental health problems in preschool children using the Strengths and Difficulties Questionnaire, which is used worldwide as a behavior‐based screening tool for assessing mental health risks, and examined its relevance to amplitude and latency MMN. As a result, we found that children at a higher risk of mental health problems had smaller MMN amplitudes than those at lower risk. It was also found that MMN amplitude was negatively correlated with the assessed higher risk of mental health problems. Although it is not clear what neural mechanisms underlie the functional association between MMN and risk of mental health problems in preschool children, the findings of this study indicate that there is an involvement of individual differences in auditory processing in childhood mental health problems. The findings suggest that such neurological changes may be prodromal symptoms of the onset of psychiatric disorders and applicable as endophenotypic markers for the early detection of various psychiatric disorders.

## Abstract 2

This study examined the relationship between mismatch negativity (MMN) during the passive oddball task and clinical assessment using a behavioral scale in nonclinical preschool children to identify neurobiological endophenotypes associated with the risk of developing mental health problems. We found that children at a higher risk of mental health problems had smaller MMN amplitudes than those at lower risk. It was also found that MMN amplitude was negatively correlated with the assessed higher risk of mental health problems.

## Graphical abstract

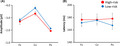

## 1 INTRODUCTION

According to a recent meta‐analysis, the prevalence of any mental disorder in children and adolescents, worldwide, is estimated to be 13.4%.^1^ Previous longitudinal studies have reported that mental health problems in early life stages increase the risk of developing mental disorders in later life stages^2^ and that mental disorders in adulthood often accompany mental health problems during adolescence.^3^ However, not all children who exhibit signs sufficient to meet the diagnostic criteria for psychiatric disorders receive appropriate medical treatment and support.^4^ These treatment‐naïve populations in childhood are at a higher risk for later psychiatric disorders, and recent studies have pointed out the possibility that the untreated periods are longer in early‐onset mental disorders.^5^

It is desirable to identify subclinical individuals exhibiting signs and at a high risk of developing mental health problems by establishing effective screening methods for timely preventive interventions. It has been pointed out that alterations of mismatch negativity (MMN), an event‐related potential (ERP) component, based on brain response to the detection of changes in auditory information (differences between input stimulus and the sensory memory of preceding stimulus), may be endophenotypes or biomarkers of the risk of developing psychiatric disorders.^6^ Establishing effective endophenotypes or biomarkers for subclinical individuals to prevent the onset of later mental disorders should be prioritized to provide objective and cost‐effective screening.^7^, ^8^ Moreover, these can be applied to the monitoring of treatment effects and the development of new treatment methods and provide new insights into the association between genetic vulnerability and the clinical phenotype of mental health problems.^7^, ^8^

The Strengths and Difficulties Questionnaire (SDQ) is one of the most frequently used questionnaires worldwide for screening mental health and difficulties in childhood and adolescence.^9^ It is a brief questionnaire comprising 25 questions and has been translated into more than 80 languages.^10^ A review of 48 studies of school‐aged children, screened using the SDQ, found that internal consistency, test‐retest reliability, and inter‐rater agreement were satisfactory for the parent and teacher versions.^9^ Regarding validity, it has been confirmed that it has a five‐factor structure; correlations with other measures of child psychopathology were high; and evidence for the screening ability of the SDQ was convincing.^9^ Furthermore, the relationship between the SDQ assessment and the prognosis has been investigated by longitudinal studies, and it has been suggested that childhood SDQ scores are significantly associated with subsequent mental health.^11^, ^12^, ^13^, ^14^, ^15^, ^16^, ^17^ However, the neural basis for various subclinical symptoms associated with the SDQ, especially in preschool children, remains insufficiently investigated.

The purpose of this study was to examine neurobiological endophenotypes associated with the risk of developing mental health problems in preschool children for the early detection of mental health problems. We assessed the risk of developing mental health problems in preschool children, using the SDQ—used worldwide as a behavior‐based screening tool for assessing mental health risks—and examined its relevance to MMN during passive oddball tasks. Our main hypothesis is that children at high risk of developing mental health problems have lower amplitude and longer latency of MMN than children at low risk, and the increased risk is negatively correlated with the amplitude and latency of MMN, as suggested by previous studies.^18^, ^19^, ^20^

## 2 METHODS

### 2.1 Participants

Forty‐three preschool children aged four to five years (23 boys, 20 girls; mean age: 60.0 ± 5.3 months; age range: 49‐69 months), recruited from public nursery schools in Fukui prefecture via advertisements, participated in the study. All children were confirmed as being typically developed by a licensed clinical psychologist during the study period. None were confirmed to have had any developmental difficulties at the age of three. The race/ethnicity of all the children was Japanese. The exclusion criteria for the participants were as follows: physical problems; diagnosis of any otorhinolaryngology disorder within the last 6 months; subjects who had undergone treatment with antiallergic agents; past diagnosis of any psychiatric or neurodevelopmental disorder; head trauma with loss of consciousness; any history of epilepsy; considerable fetal exposure to alcohol or drugs; and perinatal complications, such as premature birth (gestational age <37 weeks, birth weight <2500 g). All children that participated in the experiment had no reported vision or hearing problems, previous brain injuries, known genetic disorders, or other neurological disorders. Handedness was assessed using the Edinburgh Handedness Inventory.^21^

### 2.2 Assessment of clinical symptoms

Behavioral characteristics and psychotic symptoms were assessed using the Strengths and Difficulties Questionnaire (SDQ) for preschool‐aged children. Parents completed the SDQ,^11^, ^22^ a 25‐item questionnaire, to assess children's internalizing and externalizing behavior problems as well as prosocial behavior tendencies. The SDQ is a brief behavioral screening questionnaire for 3‐ to 16‐year‐olds. It exists in several versions to meet the needs of researchers, clinicians, and educationalists. All versions of the SDQ examine 25 attributes, some positive, and others negative. These 25 items are divided into five subscales: 1) emotional symptoms, 2) conduct problems, 3) hyperactivity/inattention, 4) peer relationship problems, and 5) prosocial behavior. It was also suggested that these subscales can be combined into groups for higher phenotypes by adding two categories: “internalizing problems” (emotional symptoms and peer relationship problems) and “externalizing problems” (conduct problems and hyperactivity/inattention).^11^ To examine the association between risk to mental health and ERP components, participants were divided into two groups, high risk and low risk, according to the total difficulty score assessed by the SDQ. Based on the criteria of the Japanese version of the SDQ,^23^ children with a cutoff value of 13 points or above were classified as "borderline" or "abnormal" and were included in the high‐risk group, and those with 12 points or below were classified as “normal” and were included in the low‐risk group.

### 2.3 MMN paradigm

The MMN components were measured from ERP induced using the auditory oddball task based on a paradigm from previous research.^24^, ^25^ Participants were presented with 1000 auditory stimuli consisting of 850 (85%) standard tones of 1000 Hz at 50 ms and 150 (15%) deviant tones of 2000 Hz at 50 ms, with a rise and fall time of 5 ms The interval between each stimulus was 600 ms The tone stimuli were presented at 75db through a speaker situated behind the participant, while participants were asked to ignore the sound and watch a silent movie animation presented by the forward visual display. The task was only to passively listen to the auditory stimuli, and no behavioral response to the stimuli was required.

### 2.4 ERP recording

Electroencephalogram (EEG) data were measured using Ag/AgCl electrodes (Nihon Kohden) at five sites according to the 10‐20 system of electrode placement. The electrodes were attached to an elastic electrode cap that was fastened with a chin strap. Horizontal electro‐oculograms (EOGs) were recorded from electrodes placed at the outer canthus of each eye, and vertical EOGs were recorded from electrodes positioned above and below the left eye. Electrodes on bilateral ears were used as a common reference. The electrodes were attached after lightly cleaning the skin to maintain an impedance level below 10 kΩ. The EEG data were recorded by a biological amplifier (Polymate II AP216; TEAC Corp., Japan) with VitalTracer software (KISSEI COMTEC Co., Ltd., Japan). The data were preprocessed by a band‐pass filter of 0.16‐100 Hz and digitized at a sampling rate of 500Hz. Filters were set at a low cutoff of 0.53 Hz and a high cutoff of 30 Hz. The recordings were notch‐filtered offline at 60 Hz.

### 2.5 ERP data

EEG data were analyzed using EPLYZER II software (KISSEI COMTEC Co., Ltd., Japan). EEG data were preprocessed to remove artifacts due to eye movements using EOG signals and then segmented into stimulus‐locked epochs of 600 ms duration (100 ms pre‐stimulus to 500 ms post‐stimulus). Across all conditions, the average number of epochs accepted after artifact exclusion was 831.2 ± 139.1 for the low‐risk group and 807.6 ± 115.4 for the high‐risk group and there was no significant difference between the groups in the number of epochs accepted (*t*(41) = 0.51, *P =*.616). All epochs were baseline‐corrected with a mean amplitude of 100 ms pre‐stimulus. MMN was measured from the difference waveform obtained by subtracting the ERP waveform of standard tones from the ERP waveform of deviant tones. The peak point for identifying the amplitude and latency of MMN was measured as the most negative point within the 100‐250 ms window of the post‐stimulus. Thus, the amplitude and latency of MMN under each tone condition (standard, deviant) were obtained for the three central and two temporal electrode sites (Fz, Cz, Pz, T3, and T4) and compared between groups (high risk, low risk).

### 2.6 Statistical analyses

All variables were tested for normal distribution using the Kolmogorov‐Smirnov test. Demographics variables were tested using chi‐squared and t tests, including age, sex, and handedness. Mixed factorial analysis of covariance (ANCOVA) compared the groups on MMN amplitude and latency. Two‐way ANCOVA was conducted for each MMN variable (amplitude and latency), with electrode site as the within‐subjects variable and group (high risk, low risk) as the between‐subjects variable. Pearson's product‐moment correlation coefficients were used to examine the relationships between the MMN and SDQ scores in both groups. The significance level was set at *P* <.05. Statistical analyses were conducted using IBM SPSS Statistics for Windows version 26.0. Armonk, NY: IBM Corp.

## 3 RESULTS

### 3.1 Demographic characteristics

The demographic and clinical characteristics of the study participants are shown in Table 1. No significant differences were found in the demographic variables, and the two groups were well matched in age, sex, handedness, and birth weight. Compared to the low‐risk group, the high‐risk group showed higher clinical symptoms in the SDQ score (Total: t[41] = 6.45, *P* <.001; Internalizing problems: t[41] = 5.94, *P* <.001; Externalizing problems: t[41] = 5.31, *P* <.001).

**TABLE 1:** Demographic and clinical characteristics of low‐risk and high‐risk groups

Measures	Low risk	High risk	Statistics	*P*‐value
Participants (n)	32	11		
Age (years)	4.62 (0.49)	4.55 (0.52)	*t*(41) = 0.62	.542
Sex (n, male/female)	17/15	6/5	*χ*(1) = 0.01	.935
Handedness (n, right/left)	29/3	10/1	*χ*(1) = 0.01	.978
Birth weight (*g*)	3024.69 (276.63)	2989.09 (381.98))	*t*(41) = 0.33	.741
SDQ Total	7.28 (3.30)	18.27 (7.98)	*t*(41) = 6.45	<.001
SDQ internalizing problems	3.06 (1.48)	8.18 (4.26)	*t*(41) = 5.94	<.001
SDQ externalizing problems	4.22 (2.61)	10.09 (4.46)	*t*(41) = 5.31	<.001

Numbers in parentheses represent standard deviations.

### 3.2 MMN amplitude and latency

**FIGURE 1:**
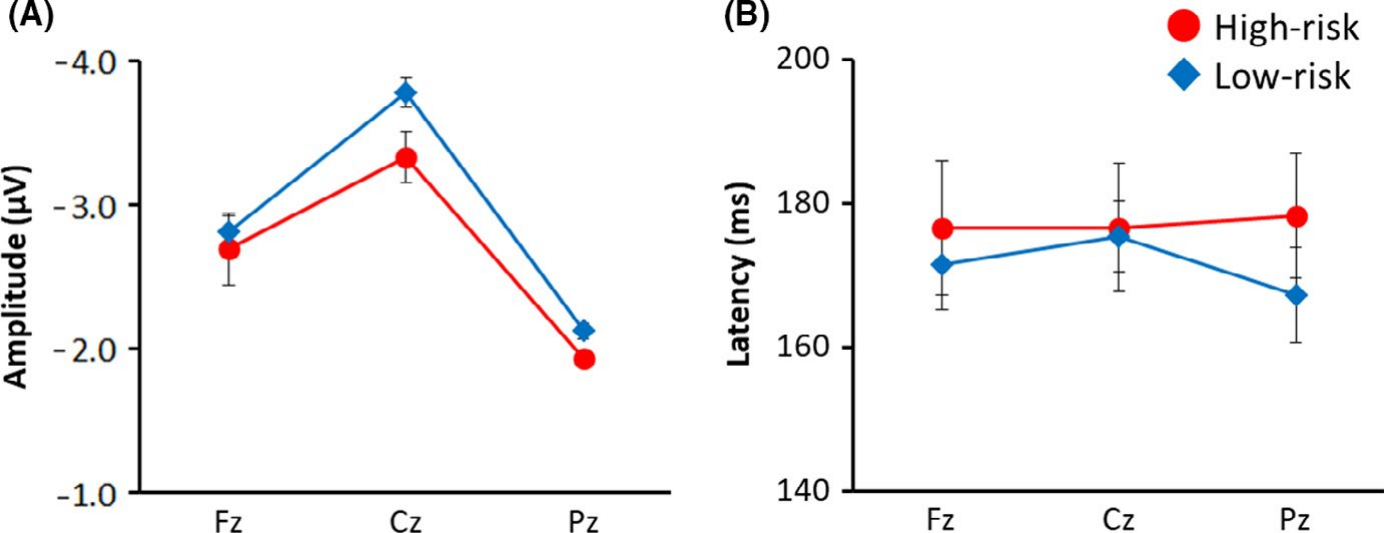
Group differences in MMN amplitude (A) and latency (B) in high‐risk and low‐risk children. Error bars denote standard error

Means and standard deviations of MMN amplitude and latency at the different electrode sites by risk group are shown in Table 2. The EEG data obtained from the temporal region (T3, T4) were often incomplete and were excluded from the analysis. Next, to examine the difference between groups of MMN amplitude, a repeated‐measures ANCOVA was performed on the amplitude with electrode site (Fz, Cz, Pz) as the within‐subjects factor and risk group (high risk, low risk) as the between‐subjects factor. There was a significant main effect of risk group on MMN amplitude (F(1,39) = 5.00, *P = *.031) and a marginally significant main effect of electrode site (F(2,78) = 2.75, *P = *.070) after controlling for age and sex (Figure 1A). The interaction between the factors was not significant (F(2,78) = 1.11, *P = *.336). Similarly, as a result of performing ANCOVA for the MMN latency with electrode site and risk group, neither the main effect nor the interaction was significant (F(1,39) = 0.01; F(2,78) = 1.05; F(2,78) = 0.50; *p*s > .10; Figure 1B). These results suggest that a higher risk of mental health problems is associated with a lower amplitude rather than latency alterations in MMN.

**TABLE 2:** Average MMN amplitude and latency in high‐risk and low‐risk children

	Amplitude (*µA*)		Latency (*ms*)	
Electrode site	Low risk	High risk	Low risk	High risk
	(n = 32)	(n = 11)	(n = 32)	(n = 11)
Fz	−2.81 (0.61)	−2.69 (0.86)	171.4 (6.2)	176.5 (9.3)
Cz	−3.77 (0.49)	−3.33 (0.62)	175.4 (4.9)	176.6 (8.9)
Pz	−2.12 (0.30)	−2.07 (0.28)	167.3 (6.5)	178.3 (8.6)

The numbers in parentheses denote standard error.

### 3.3 Relationship between MMN amplitude and behavioral adaptation levels

**FIGURE 2:**
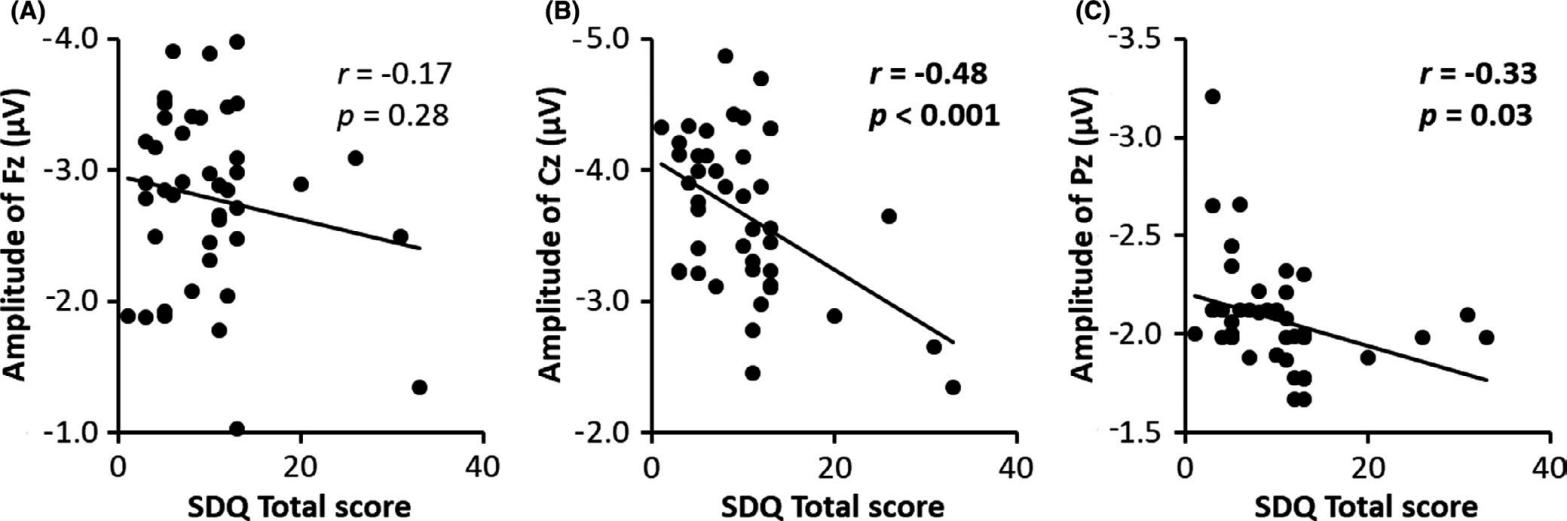
Correlations between the amplitudes of MMN and the SDQ total score (A: Fz, B: Cz, C: Pz)

To investigate the relationship between atypical neural response and behavioral risk of mental health problems in children, we performed a correlation analysis with the SDQ total score and MMN amplitudes observed for their attenuation in the high‐risk group. The results indicated a significant negative correlation with the SDQ total score at Cz and Pz (Cz: *r* = −0.48, *P < *.001; Pz: *r* = −0.33, *P = *.03; Figure 2B,C), whereas no correlation was observed for Fz (*r* = −0.33, *P = *.03; Figure 2A). These results suggest that a higher risk of mental health problems is associated with lower MMN amplitudes at Cz and Pz. Next, when the relationship with the SDQ subscale was examined, a significant negative correlation was confirmed between the externalizing problems and both Cz and Pz (Cz: *r* = −0.50, *P < *.001; Pz: *r* = −0.38, *P = *.01), but the internalizing problems only correlated with Cz (*r* = −0.37, *P = *.01).

## 4 DISCUSSION

This study investigated the relationship between MMN during the passive oddball task and clinical assessment using the behavioral scale in preschool children to identify neurobiological endophenotypes associated with the risk of developing mental health problems. As a result, we found that children at a higher risk of mental health problems had smaller MMN amplitudes than those at lower risk. It was also found that MMN amplitude was negatively correlated with the assessed higher risk of mental health problems. Although it is not clear what neural mechanisms underlie the functional association between MMN and risk of mental health problems in preschool children, the findings of this study indicate that there is an involvement of individual differences in auditory processing in childhood mental health problems. Individual variations in the early stages of auditory information processing may affect subsequent processing, leading to individual differences in perceptual properties of acoustic stimuli.^26^, ^27^ Another possibility is that top‐down processes are involved in various stages of the auditory processing pathway. Thus, although the findings of this study could not clarify the neural basis of functional associations, individual differences in certain types of sensitivity to acoustic information in preschool childhood could be available as an endophenotype for predicting the risk of developing mental health problems.

To the best of our knowledge, this is the first study to investigate the relationship between MMN characteristics and the risk of mental health problems in a community sample of preschool children. As mentioned above, MMN is induced in the frontal cortex, in association with attentional shift based on the automatic detection of changes in auditory stimuli.^6^, ^28^ MMN is an ERP component of preattentive processing, and it has been suggested that MMN is involved in preattentive cognitive processing.^29^ In the context of mental health problems, it has been reported that the amplitude of MMN is attenuated in children with attention‐deficit/hyperactivity disorder (ADHD), compared with the control‐group children.^19^, ^30^, ^31^ It has also been indicated that MMN alterations are associated with several psychiatric disorders, such as schizophrenia, bipolar disorder, and major depression.^32^, ^33^, ^34^, ^35^ In addition, MMN has been suggested as an indicator of cognitive decline observed in most psychiatric disorders.^36^ The results of the present study are consistent with these findings. In this study, we found that the reduction in MMN amplitude at Cz and Pz was associated with behavioral symptoms in mental health problems, as measured by the SDQ scores, suggesting that MMN amplitude may be a precursor indicator for detecting abnormalities in cognitive function in preschool children at risk of developing mental health problems.

It is an interesting question as to what symptoms in psychiatric health problems are specifically associated with reduced MMN amplitude. In this study, externalizing problems were correlated with both Cz and Pz amplitudes on the SDQ subscale, while internalizing problems were only correlated with Cz amplitude. Although hyperactivity/inattention is a major component of externalizing problems, previous studies have reported that children with ADHD not only have reduced MMN amplitude, but their severity, including both hyperactivity and inattention, is also negatively correlated with Pz amplitude.^19^, ^31^ On the other hand, reduced MMN has also been suggested in association with internalizing problems in children.^20^, ^37^ Previous studies have reported that children with socio‐emotional difficulties, such as autism spectrum disorders and social withdrawal, have lower MMN amplitudes in the frontal lobe including Fz and Cz electrodes, especially in Cz.^20^, ^37^ While the authors note that the functional association between MMN and socio‐emotional difficulties is not clear, these findings point to the involvement of individual differences in the sensitivity and resolution of auditory signals in childhood social interaction. Taken together, while the decrease in MMN amplitude reflects the cognitive decline commonly seen in psychiatric disorders, the relative difference in MMN amplitude may reflect each spectrum of symptoms. In other words, externalizing problems, such as hyperactivity/inattention, may be reflected in Pz as greater attenuation, while internalizing problems, such as socio‐emotional introversion, may be reflected in Cz.

It should also be noted that we could not find any significant differences in the amplitude at the Fz or the latency of all the electrodes. First, regarding the Fz amplitude, previous studies have robustly shown a decrease in Fz amplitude relative to MMN in an adult population with schizophrenia,^18^, ^28^ whereas an increase in amplitude has been reported in children at risk of developing schizophrenia.^32^ Although the risk of mental health problems was assessed using SDQ in the current study, it may not have been sufficiently sensitive with regard to the risk of psychosis (such as can be found with schizophrenia) and thus not have been reflected as a difference in amplitude at Fz. Next, with respect to latency, while several studies have reported longer latency in MMN, the association with psychiatric states at each electrode is inconsistent compared to the findings on amplitude.^18^, ^19^, ^38^ It is plausible that no significant differences were found in latency in this study because the previous studies assessed psychiatric populations, whereas this study assessed the at‐risk population. Thus, the latency of MMN may not be suitable for use as a precursor index for risk assessment of mental health problems due to insufficient sensitivity.

Several limitations of the present study should be noted and taken into consideration in future studies. First, this study included a relatively small sample of participants and utilized a cross‐sectional design that precluded the identification of causal links between the risk of developing mental health problems and brain functions as their neural basis. Longitudinal studies utilizing larger sample sizes are required to elucidate the association more fully between MMN as an endophenotype and the risk of developing mental health problems. Second, our study assessed the risk of developing mental health problems through behavioral screening using a single scale by a single informant (SDQ teacher version). SDQ assessments by multiple informants have been reported to be more sensitive, and future studies employing other neuropsychological tests and/or assessing by multiple informants would be helpful in strengthening our hypothesis. Finally, children's cognitive abilities based on intelligence or developmental quotients were not controlled in group comparisons in this study. Therefore, it was difficult to distinguish whether reduced MMN was associated with alterations in specific attentional function or general cognitive function. Although we confirmed through the exclusion criteria that the children had never been diagnosed with developmental problems, appropriate cognitive assessment should be performed to distinguish between the two in future studies. Despite these limitations, this study clarifies the neurobiological endophenotypes associated with the risk of developing mental health problems in preschool children.

In conclusion, in the general population of preschool children, we found that children at a higher risk of developing mental health problems had reduced MMN amplitudes in the frontal and parietal lobe regions, compared to those at a lower risk. Since such neurological changes may be prodromal symptoms of the onset of psychiatric disorders, they may be applicable as endophenotypic markers for the early detection of various psychiatric disorders. In the future, it is important to clarify how the association between the identified neurological changes and the risk of mental illness follows in subsequent development, especially in adolescence.

## CONFLICT OF INTEREST

The authors declare no conflicts of interest.

## AUTHOR CONTRIBUTIONS

TA and AT conceived the project and designed the experiments. TA performed the experiments and collected the data. TA and TXF analyzed the data. All authors wrote the manuscript and approved the final manuscript.

## APPROVAL OF THE RESEARCH PROTOCOL BY AN INSTITUTIONAL REVIEWER BOARD

The study protocol was approved by the Research Ethics Committee of the University of Fukui, Japan (Assurance No. FU24‐82), and all procedures were conducted following the Declaration of Helsinki and the Ethical Guidelines for Clinical Studies of the Ministry of Health, Labour, and Welfare of Japan.

## INFORMED CONSENT

All parents of the participants provided written informed consent for participation in this study.

## Data Availability

The data cannot be made publicly available as data sharing was not included in the consent.
